# miR-32 promotes MYC-driven prostate cancer

**DOI:** 10.1038/s41389-022-00385-8

**Published:** 2022-03-01

**Authors:** Mauro Scaravilli, Sonja Koivukoski, Andrew Gillen, Aya Bouazza, Pekka Ruusuvuori, Tapio Visakorpi, Leena Latonen

**Affiliations:** 1 Institute of Biomedicine, University of Eastern Finland, Kuopio, Finland; 2 Institute of Medical Sciences, School of Medicine, Medical Sciences and Nutrition, University of Aberdeen, Scotland, UK; 3 Institute of Biomedicine, University of Turku, Turku, Finland; 4 Faculty of Medicine and Health Technology, Tampere University and Tays Cancer Center, Tampere University Hospital, Tampere, Finland; 5 Fimlab Laboratories Ltd, Tampere University Hospital, Tampere, Finland

**Keywords:** Prostate cancer, Non-coding RNAs, Oncogenes, Cancer models

## Abstract

miR-32 is an androgen receptor (AR)-regulated microRNA, expression of which is increased in castration-resistant prostate cancer (PC). We have previously shown that overexpression of miR-32 in the prostate of transgenic mice potentiates proliferation in prostate epithelium. Here, we set out to determine whether increased expression of miR-32 influences growth or phenotype in prostate adenocarcinoma in vivo. We studied transgenic mice expressing MYC oncogene (hiMYC mice) to induce tumorigenesis in the mouse prostate and discovered that transgenic overexpression of miR-32 resulted in increased tumor burden as well as a more aggressive tumor phenotype in this model. Elevated expression of miR-32 increased proliferation as assessed by Ki-67 immunohistochemistry, increased nuclear density, and higher mitotic index in the tumors. By gene expression analysis of the tumorous prostate tissue, we confirmed earlier findings that miR-32 expression regulates prostate secretome by modulating expression levels of several PC-related target genes such as *Spink1*, *Spink5*, and *Msmb*. Further, we identified *Pdk4* as a tumor-associated miR-32 target in the mouse prostate. Expression analysis of *PDK4* in human PC reveals an inverse correlation with miR-32 expression and Gleason score, a decrease in castration-resistant and metastatic tumors compared to untreated primary PC, and an association of low *PDK4* expression with a shorter recurrence-free survival of patients. Although decreased PDK4 expression induces the higher metabolic activity of PC cells, induced expression of PDK4 reduces both mitotic respiration and glycolysis rates as well as inhibits cell growth. In conclusion, we show that miR-32 promotes MYC-induced prostate adenocarcinoma and identifies PDK4 as a PC-relevant metabolic target of miR-32-3p.

## Introduction

Prostate cancer (PC) is the most common cancer and the third leading cause of cancer death in men in developed countries [1]. Localized PC can be cured with prostatectomy and/or radiation therapy. The growth of PC is driven by androgens, and thus, androgen deprivation therapy (ADT) is the primary form of treatment in advanced disease. ADT will unequivocally lead to the emergence of, so-called castration-resistant prostate cancer (CRPC), which is a highly aggressive form of the disease with a poor prognosis [2]. Although the use of prostate-specific antigen (PSA) for screening of asymptomatic men for PC has reduced disease-specific mortality, screening is associated with overdiagnosis and additional markers are required for PC diagnosis and prognosis [3]. Furthermore, novel therapeutic targets are required, underlining the importance of understanding molecular mechanisms of especially CRPC.

Micro-RNAs (miRNAs) are non-coding RNA molecules of 22 nucleotides (nt). MiRNAs regulate their target RNAs by binding to a complementary or near-complementary sequence, often residing in the 3′-end untranslated region (3′-UTR) region of messenger RNAs (mRNAs) [4]. This binding leads to either degradation of the mRNA or, alternatively, to suppression of translation of the mRNA [5]. Since one miRNA can have multiple targets and a particular mRNA can be targeted by several miRNAs, miRNAs are parts of complex networks regulating gene expression [6, 7]. Several miRNAs are deregulated during the formation of cancer, including PC, influencing several cancer-related cellular functions, such as androgen signaling, cell cycle, DNA repair, cell adhesion, migration, invasion, and regulation of apoptosis [8–11].

Several studies have assessed alterations of miRNA expression levels in PC [12–19] and their potential role as PC biomarkers has been recognized [20–22]. Furthermore, several individual miRNAs have functional importance in PC [8, 23]. We [24] and others [25, 26] have shown that miR-32 is one of the most consistently deregulated miRNAs in PC, with an expression of miR-32 increased especially at the CRPC stage. In PC cells in vitro, elevated expression of miR-32 increases proliferation and decreases apoptosis [24, 27]. We have also shown that the expression of miR-32 is regulated by androgens, the major driver of the disease [24, 28]. By overexpressing miR-32 tissue specifically in the prostate of transgenic mice, we previously showed that miR-32 induces proliferative and metaplastic alterations in the prostate epithelium, as well as regulates the expression of several secreted factors [29]. Transgenic overexpression of miR-32 increases the expression of proliferation markers in prostate epithelium. In *Pten* heterozygous background, the incidence of prostatic intraepithelial neoplasia (PIN) is slightly increased by transgenic overexpression of miR-32 [29]. However, no aggressive tumors were detected in these previous models, and thus the biological effects of increased miR-32 expression in prostate carcinoma remain unstudied.

To better understand the possible role of miR-32 in PC, we wanted to assess the physiological effects of increased expression of miR-32 in PC in vivo. For this, we utilized transgenic mice expressing MYC oncogene (hiMYC mice) [30] to induce tumorigenesis in the mouse prostate and studied the effects of transgenic overexpression of miR-32 in this model. Our findings show that miR-32 promotes Myc-induced prostate adenocarcinoma by increasing the volume and aggressive phenotype of the tumors. We also identify transcriptomic changes induced by miR-32 in tumor-containing prostates and identify PDK4 as a target of miR-32 relevant for human PC.

## Results

### Transgenic expression of miR-32 results in increased tumor load and a more aggressive phenotype in prostate tumors of hiMYC mice

We have previously described the ARR2PB-miR32 transgenic mouse line overexpressing miR-32 specifically in the prostate post puberty under an AR-responsive promoter [29]. Here, we cross-bred these mice with the hiMYC model mice overexpressing oncogenic MYC prostate-specifically, thereby inducing prostate adenocarcinoma with high penetrance [30]. First, we confirmed the expression of both transgenes in the prostates of the mice at 1 and 3 months of age (Supplementary Fig. 1A–D). As previously reported, transgenic miR-32 is expressed in the ventral (VP) at the highest level, with lateral (LP) and dorsal (DP) having moderate expression (Supplementary Fig. 1A), while transgenic MYC is expressed in VP, LP, and DP lobes equally (Supplementary Fig. 1C). Neither of the transgenes significantly affects each other’s expression levels (Supplementary Fig. 1B, D).

**Fig. 1:**
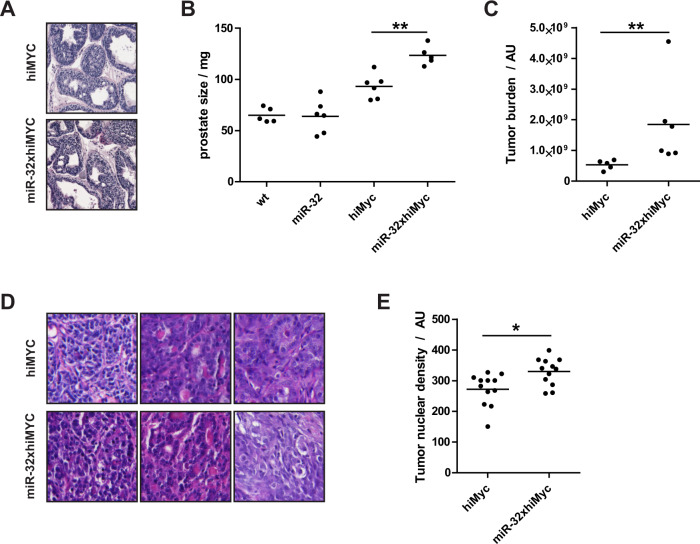
Transgenic miR-32 promotes tumor development in hiMYC model of prostate adenocarcinoma. **A** Histology of prostate shows intraepithelial neoplasia at 1 month of age in both hiMYC and miR-32xhiMYC mice. Examples from HE-stained lateral lobe. **B** Prostate size at 6 months of age in wt, miR-32 transgenic, hiMYC, and miR-32xhiMYC mice. **C** Tumor burden quantified as a sum of tumor areas on whole slide images of tissue sections taken every 50 µm apart throughout the organ in prostates of hiMYC and miR-32xhiMYC mice at 6 months of age. **D** Example histology of tumors in 6-month-old hiMYC and miR-32xhiMYC mouse prostates. **E** Tumor nuclear density in most advanced tumors in 6-month-old hiMYC and miR-32xhiMYC mouse prostates. *p* values *<0.05, **<0.01.

Histological examination shows that, as previously described, the prepubertal (1mo) prostate epithelium is already hyperproliferative in the hiMYC mice (Fig. 1A), whereas no apparent difference in the histology of the prostate is detectable by added miR-32 transgene (Fig. 1A). At 6 months of age, the MYC-expressing prostates have well-developed tumors of adenocarcinoma [30], which is reflected in the size of the prostates of the hiMYC mice compared to wt mice (Fig. 1B). Interestingly, transgenic expression of miR-32 induces a further, statistically significant increase in prostate size in the hiMYC background (Fig. 1B). As the tumorous prostates at this age often harbor thickened stroma, inflammation, and promoted vasculature, we wanted to analyze whether the size of miR32xhiMYC prostates results from such changes or larger tumors. We performed quantitation of tumor areas from histological sections throughout the prostate including neoplastic tumorous areas and excluding stroma, large veins, and large vacuoles. This analysis revealed that the tumors in miR-32-overexpressing prostates were significantly larger than in the hiMYC-only prostates (Fig. 1C). In general, the variance in the histology of miR-32xhiMYC tumors was similar to hiMYC tumors at 6 months of age when the tumors have developed to adenocarcinoma (Fig. 1D). Yet, 2/6 miR-32xhiMYC mice showed signs of local invasion perivascularly and outside the normal prostate gland structures surrounded by basal and smooth muscle layers (Supplementary Fig. 2A). In addition, occasional highly infiltrative tumor growth patterns were detected in the miR-32xhiMYC mouse tumors at 6 months of age (Supplementary Fig. 2B). These results indicate that miR-32 tumors have a slightly increased rate of development and signs of more aggressive phenotypes. As a further marker for increased aggressiveness, we quantified tumor nuclear density in most developed tumor areas in mice of 6 months of age and detected that miR-32-overexpressing tumors had significantly increased nuclear density compared to hiMYC-only tumors (Fig. 1E). These data show that miR-32 promotes MYC-induced tumorigenesis in the mouse prostate.

### Molecular marker analysis shows increased replication and mitotic activity in miR-32 overexpressing, MYC-induced adenocarcinoma

**Fig. 2:**
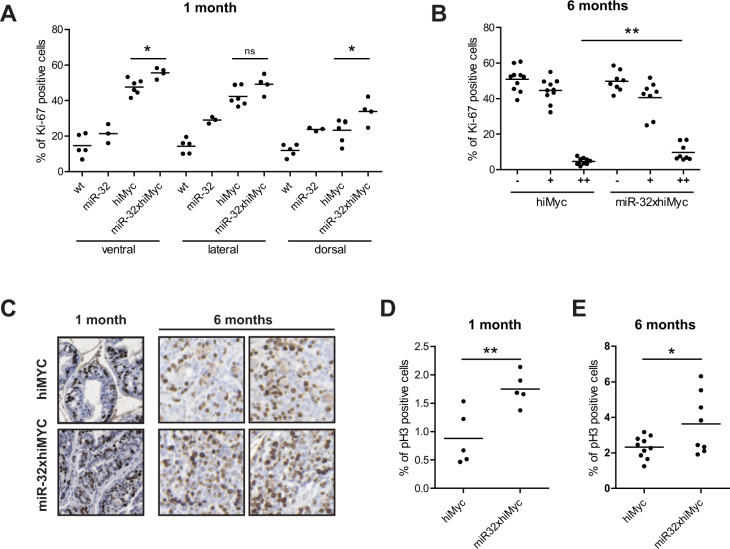
Proliferation is induced by transgenic miR-32 in prostate epithelium and tumors in hiMYC mice. **A** Percentage of prostate epithelial nuclei positive for proliferation marker Ki-67 immunostaining in ventral, lateral, and dorsal lobes of wt, miR-32 transgenic, hiMYC, and miR-32xhiMYC mice at 1 month of age. **B** Percentage of nuclei positive for Ki-67 immunostaining in hiMYC and miR-32xhiMYC mice tumors at 6 months of age. – no staining, + weak to intermediate staining, ++ strong staining. **C** Example images of Ki-67 immunostaining in hiMYC and miR-32xhiMYC mouse prostate epithelium at 1 month and tumors at 6 months of age. **D** Percentage of prostate epithelial cells positive for mitotic marker pH3 immunostaining in hiMYC and miR-32xhiMYC mice at 1 month of age. **E** Percentage of cells positive for pH3 immunostaining in hiMYC and miR-32xhiMYC mice tumors at 6 months of age. *p* values *<0.05, **<0.01.

To study the cellular mechanism associated with the increased tumor load in miR-32 overexpressing prostates, we studied whether transgenic miR-32 expression affects the rates of proliferation, mitosis, or apoptosis in the MYC-induced adenocarcinoma. We performed an immunohistochemical analysis in both the prepubertal epithelium (at 1 month) and the hiMYC-induced tumors (at 6 months). The proliferative index of the prepubertal, premalignant epithelium, determined as the percentage of cells staining positive for proliferation marker Ki-67, was higher in the hiMYC prostates than the wt or miR-32 only prostates, and expression of miR-32 increased the proliferation index further (Fig. 2A, Supplementary Fig. 2A). In the tumors, the overall percentage of Ki-67-positive cells was not significantly different between hiMYC and hiMYCxmiR-32 tumors (data not shown), but hiMYCxmiR-32 tumors displayed a significantly increased percentage of high-intensity nuclear staining (Fig. 2B, Supplementary Fig. 2B). The mitotic rate, as measured by the percentage of cells staining immunohistochemistry (IHC) positive for phosphorylated histone 3 (pH3), was significantly higher in both premalignant epithelium and in tumors of the double-transgenic mice as compared to the hiMYC-only mice (Fig. 2C, D). While there were no significant differences detected in the apoptotic index, as measured by the percentage of cells staining IHC positive for activated caspase-3 (Casp3), either in the premalignant prostate epithelium or in the tumors (Supplementary Fig. 2C, D), a non-significant trend towards decreased Casp3 staining and, thus, decreased apoptotic rate was visible in the premalignant stage (Supplementary Fig. 2C). These results demonstrate that miR-32 promotes MYC-induced tumorigenesis by increasing the proliferation and mitotic rate both during tumor development and in the developed tumors.

### Gene expression analysis of high miR-32-expressing tumors identifies PDK4 as a PC-relevant miR-32 target

**Fig. 3:**
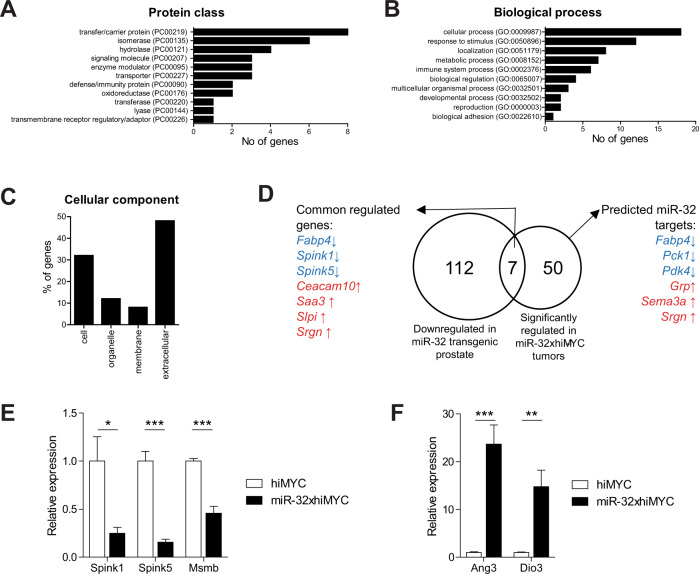
Effects of transgenic miR-32 on gene expression in hiMYC-induced prostate cancer in mice. Gene expression analysis was performed on miR-32xhiMYC compared to hiMYC mouse tumorous prostates at 6 months of age. **A** Protein class distribution, **B** biological processes, and **C** cellular component of genes differentially expressed in miR-32xhiMYC compared to hiMYC mice. **D** Venn diagram showing common genes between genes downregulated in mouse prostate by transgenic miR-32 expression from a previous study [29] and genes differentially expressed in tumorous mouse prostates of miR-32xhiMYC compared to hiMYC mice in this study. The identified commonly regulated genes are shown, as well as genes that are predicted or verified targets for miR-32 among the differentially expressed genes in miR-32xhiMYC compared to hiMYC mice in tumorous prostates. **E** RT-qPCR validation of genes downregulated by transgenic miR-32 expression. **F** RT-qPCR validation of genes upregulated by transgenic miR-32 expression.

To understand the gene regulatory network through which miR-32 exerts its tumor-promoting effects in the prostate, we studied gene expression differences between hiMYC and hiMYCxmiR-32 mouse prostates at 6 months. RNA was extracted from sections of tumorous prostates and subjected to microarray analysis. We found 57 genes to be significantly regulated >twoold (Supplementary Table 1). These included genes belonging to several biological processes and protein classes, most prominently proteins with enzymatic functions and roles in multiple forms of cellular transport (Fig. 3A, B). Interestingly, nearly half of the regulated genes are defined as extracellular, indicating that miR-32 prominently affects the secretome of prostatic tissue (Fig. 3C). Pathway analysis of the significantly regulated genes in mice and for the corresponding human orthologs indicated the involvement of especially PPAR signaling and metabolic pathways (Supplementary Fig. 3A–D, Supplementary Tables 2–5).

Comparing the list of significantly regulated genes to our previous data set of genes downregulated by transgenic overexpression of miR-32 [29], we found seven genes in common (Fig. 3D, Supplementary Table 1). Three of these genes were downregulated, including *Spink1* and *Spink5*, which we identified as prostate-relevant miR-32 targets in our previous work [29]. We used these genes and *Msmb*, a prostate-specific secretory factor known to decrease in expression in PC, to confirm the validity of our microarray approach by performing an RT-qPCR analysis for them including samples from wt, transgenic miR-32, hiMYC, and hiMYCxmiR-32 mice. The expression of both *Spink1* and *Spink5* was confirmed to decrease in response to transgenic expression of miR-32 (Fig. 3E). These differences are more visible in the non-tumorous prostates of wt and transgenic miR-32 mice, as in the hiMYC mice the levels of expression of these genes decrease significantly. *Msmb*, expression of which is also downregulated by expression of hiMYC, is increased by transgenic miR-32 alone, as compared to wt prostate (Fig. 3E). To confirm miR-32-induced gene expression changes found in our microarray data, we analyzed the expression of *Ang3* and *Dio3*, with RT-qPCR. *Ang3* expression in the prostate is increased by miR-32, especially in the non-cancerous epithelium (miR-32 only compared to wt) but not in tumors (hiMYC compared to hiMYCxmiR-32), while *Dio3* expression shows an opposite pattern (Fig. 3F).

We performed additional analysis to search for direct and clinically relevant targets of miR-32. We narrowed down our list of candidate miR-32-regulated genes by querying database information for confirmed or predicted miR-32 targets. Six genes were targets for either miR-32-5p, miR-32-3p, or both (Supplementary Table 1). Two of these genes, *Srgn* and *Fabp4*, were downregulated in the mouse prostate by miR-32 in our previous gene expression analysis. Thus, we performed RT-qPCR analysis for the expression of these genes in tumor-containing hiMYC mouse prostates in the presence or absence of transgenic miR-32, as well as queried their expression in human prostate tumor tissue data. Although *Fabp4* was confirmed to be downregulated in miR-32 overexpressing tumor tissue compared with hiMYC-only samples, the expression of *FABP4* in human PC, according to previously obtained RNA-sequencing data of our Tampere cohort of patients [31], was low and not significantly altered in CRPC compared with PC samples (data not shown). *Srgn*, on the other hand, was upregulated in miR-32 expressing hiMyc tumors, but *SRGN* downregulated in CRPC compared with PC patient tumor specimens (data not shown). Hence, neither *FABP4* nor *SRGN* represent a clinically relevant target of miR-32 in PC. Of the two other genes indicated as direct, downregulated targets for miR-32, namely Pck1 and Pdk4 (Supplementary Table 1), Pck1 is not expressed in prostate tumors of patients (data not shown) and thus it is also unlikely to represent a clinically relevant miR-32 target in PC.

**Fig. 4:**
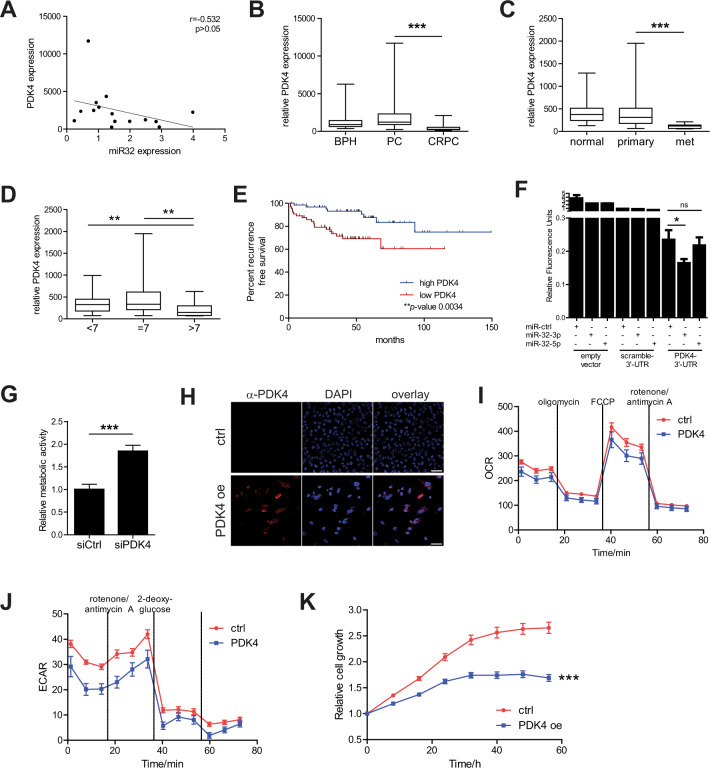
PDK4 is a prostate cancer-relevant target of miR-32. **A** Correlation analysis of PDK4 expression defined by RNA-seq [31] and miR-32 expression defined by RT-qPCR in 15 human primary PC samples show an inverse correlation between the expression of these genes. **B** RNA expression analysis in prostate tissue samples of BPH, primary cancer (PC), and CRPC in the Tampere patient cohort showed decreased PDK4 expression in CRPC compared to PC. **C** RNA expression analysis in prostate tissue samples of normal, primary cancer, and metastases including both non-castrate and castration-resistant samples in the Taylor et al. [46] patient cohort showing decreased PDK4 expression in metastatic compared to PC samples. **D** RNA expression analysis in prostate tissue samples of primary PC samples in the Taylor et al. [46] patient cohort showing relatively decreased PDK4 expression in samples with higher Gleason grades. **E** Survival proportions of patients with primary PC in the Taylor et al. [46] data set between tumors of high and low PDK4 expression show decreased recurrence-free survival for patients with low PDK4-expressing tumors. **F** Luciferase assay in HeLa cells transfected with control (no-UTR, scramble-3′-UTR) and PDK4-3′UTR luciferase constructs and the indicated pre-miRNAs showing targeting of PDK4-3′-UTR construct targeting by pre-miR-32-3p. **G** Downregulation of PDK4 expression increases the relative metabolic activity as defined by Alamar Blue assay of 22Rv1 PC cells. **H** Immunofluorescence analysis of PC-3 cells transiently transfected with PDK4 (PDK4 oe) compared with cells transfected with the control plasmid (ctrl). Staining with α-PDK4 antibody shows no expression of endogenous PDK4 protein in PC-3 cells and positive PDK4 signal at 3 days after transfection. DAPI nuclear staining is shown for reference. Scale bar, 50 µm. **I** Oxygen consumption rate in PC-3 cells transfected with control (ctrl) or PDK4-expressing plasmids showing decreased basal and maximum mitochondrial respiration rate in PDK4 overexpressing cells. **J** Extracellular acidification rate in PC-3 cells transfected with control (ctrl) or PDK4-expressing plasmids showing decreased basal and maximum glycolytic rates. **K** PC-3 cells transiently transfected with PDK4 (PDK4 oe) show decreased growth compared to cells transfected with the control plasmid (ctrl). Error bars, standard deviation (**F**, **G**), SEM (**I**–**K**). *p* values *<0.05, **<0.01, ***<0.001.

We further studied Pdk4, Pyruvate Dehydrogenase Kinase 4, expression of which was confirmed by RT-qPCR to be downregulated by miR-32 in hiMYC tumorous prostates (Supplementary Fig. 4A). Next, we measured the expression of miR-32 with RT-qPCR in 15 PC samples from our Tampere cohort that has previously been analyzed with RNA-seq for gene expression and found an inverse correlation between the miR-32 and PDK4 expression (Fig. 4A). A similar inverse correlation between miR-32 and PDK4 expression was observed in an independent, publicly available data set of primary prostate tumor samples (Supplementary Fig. 4B). By studying two independent data sets, we found that PDK4 expression is significantly decreased in CRPC compared to PC tumors (Fig. 4B), and in metastases compared to primary tumors in a data aset containing both non-castrate and castration-resistant tumors (Fig. 4C). PDK4 expression was also lower in primary prostate tumors of higher Gleason grade (>7; Fig. 4D), and low PDK4 expression in primary tumors was associated with a shorter recurrence-free survival of patients (Fig. 4E). To show that PDK4 was in fact a direct target of miR-32, and to determine which miR-32 forms target PDK4-3′-UTR, we performed reporter luciferase assays. We transfected HeLa cells with luciferase constructs without a 3′-UTR, with scrambled 3′-UTR, and the 3′-UTR of PDK4 in combination with either a control pre-miRNA, or pre-miR-32-3p or pre-miR-32-5p. The results demonstrate that, as expected based on target prediction, miR-32-3p targets PDK4-3′-UTR while miR-32-5p fails to do so (Fig. 4F).

To demonstrate the functional relevance of PDK4 expression in PC cells, we downregulated PDK4 expression with esiRNA, a heterogeneous pool of siRNA of natural RNA that all target the same mRNA sequence, in 22Rv1 cells, the only PC cell line with detectable levels of PDK4 expression (Supplementary Fig. 4C). The downregulation of PDK4 by siRNA was confirmed both at the mRNA and protein levels with RT-qPCR and western blotting, respectively (Supplementary Fig. 4E, F). As PDK4 is a metabolic enzyme contributing to the regulation of glucose metabolism, we performed an assay measuring the metabolic activity of the siRNA-treated cells. Figure 4G shows that the downregulation of PDK4 increases the metabolic activity of PC cells. To further test PDK4 effect on PC cell metabolism, we overexpressed PDK4 in PC-3 cells with negligible endogenous PDK4 expression (Supplementary Fig. 4C, Fig. 4H) and determined mitochondrial respiration and glycolysis rates of the cells via measuring the oxygen consumption and extracellular acidification rates (Fig. 4I, J). The results show that increased expression of PDK4 decreases both the basal and maximum mitochondrial respiration rates as well as the basal and maximum glycolytic rates in PC cells. Further, the expression of PDK4 decreases the growth of PC-3 cells (Fig. 4K). Collectively, our results show that PDK4 affects PC cell metabolism and provide a mechanistic explanation of the benefit of PDK4 downregulation in advanced prostate tumors.

## Discussion

Here, we showed that miR-32 promotes prostate tumors induced by the expression of oncogenic MYC. We found that transgenic expression of miR-32 increases proliferation, mitotic index, prostate and tumor size, and nuclear density of advanced tumors significantly in the adenocarcinoma tumors induced in the hiMYC model. Already at 1 month of age, the epithelium in the mouse prostate showed an increased level of proliferation and mitotic markers, and signs of earlier local invasion were detected. These results indicate that the expression of miR-32 contributes to both tumor development and progression in the hiMYC-induced adenocarcinoma.

Mouse prostate is known to be relatively resistant to tumor formation [32]. Thus, several genetic alterations are often required to break the tumor-forming barrier of mouse prostate epithelium. Previously, we studied the possible tumor-promoting functions of miR-32 by transgenic expression in Pten heterozygous mice, known to be susceptible to hyperplasia and high-grade PIN [29, 33]. In this genetic background, an increase in the incidence of PIN with transgenic miR-32 expression was noted, while no cancer formation was initiated. In here, we cross-bred the transgenic miR-32-expressing mice with a genetic model initiating prostate adenocarcinoma by expression of the MYC oncogene. In this background, the tumor-promoting effect of miR-32 was evident. These results demonstrate that, although miR-32 is not an oncogene capable of inducing carcinoma in the prostate, it is a context-dependent tumor promoter. While it is evident that miR-32 tumor-promoting effect can take place in the hiMYC setting, further studies are required to determine whether also other genetic settings, such as expression of other initiating oncogenes, exist where miR-32 can promote tumor development.

We have previously shown miR-32 to lower the rate of apoptosis in LNCaP PC cells in vitro [24]. In our previous in vivo study, no evidence of decreased apoptosis by transgenic miR-32 expression was found neither in normal epithelium nor in PIN lesions in the Pten+/− background. Here, a weak trend toward the lowered level of apoptosis in the prostatic epithelium in mice of 1 month of age was noted as measured with immunohistochemical analysis of activated Caspase-3 staining, but the tumors did not show a similar trend. Thus, we conclude that the tumor-promoting effect of miR-32 in the prostate results in more from an increased rate of cell proliferation rather than decreased apoptosis.

We studied the gene expression changes induced by transgenic expression of miR-32 in the prostates with tumors and found that miR-32 significantly affects prostate secretome. In this study, nearly half of the genes that we found significantly regulated in our gene expression analysis, encode for extracellular proteins. Previously, we demonstrated that overexpression of miR-32 in the prostate in vivo affects the expression of Spink1 and Spink5 [29]. Here, we confirm these findings that Spink1 and Spink5, serine protease inhibitors of Kazal-type, are downregulated by miR-32 both in normal prostate and prostate adenocarcinoma. SPINK1 is overexpressed in 5–10% of PC and is known to be associated with the aggressive disease as well as to play a role in epithelial–mesenchymal transition (EMT) in the prostate [34]. SPINK1 is transcriptionally repressed by the AR [35], and our results show that the AR-induced miR-32 supports this downregulation. Very little is known of the role of Spink5 in PC. Microseminoprotein-beta (MSMB, also called PSP94) is a major secretory product of the prostate epithelial cells. MSMB synthesis is decreased in PC [36], and the MSMB levels are reduced both in tumors and in circulation [37]. MSMB expression is influenced by androgens, but also by genotype and epigenetic silencing [38]. Our work shows that miR-32 contributes to the regulation of Msmb levels in MYC-induced adenocarcinoma of the prostate.

MiRNAs are parts of complex regulatory networks and can thus affect the expression of genes also indirectly. We wanted to identify direct miR-32 targets with importance in PC and screened for genes that were transcriptionally altered by transgenic miR-32 in the mouse tumors, predicted or verified miR-32 targets, and had significant expression alterations in human PC. With this approach, we identified PDK4 as a PC-relevant miR-32 target and showed direct regulation of PDK4-3′-UTR by miR-32-3p. PDK4 is a pyruvate dehydrogenase kinase belonging to a family of four kinases. Together with pyruvate dehydrogenase complex, the PDK isoforms 1–4 are the main regulators of the metabolic shift from oxidative phosphorylation (OXPHOS) to glycolysis, known as the Warburg effect, which is characteristic for many cancers. Pdk4 phosphorylates and inactivates pyruvate dehydrogenase, resulting in the directing of pyruvate toward lactate production rather than entry in the mitochondrial tricarboxylic acid (TCA) cycle. This metabolic shift gives the cancer cells a survival advantage in the hypoxic tumor microenvironment and protects them from cytotoxic effects of oxidative damage and apoptosis. PDKs are known to be overexpressed in several cancers and associated with bad prognosis and therapy resistance [39]. However, in contrast to other PDK isoforms, not only oncogenic, but also tumor-suppressive functions of PDK4 have been reported [reviewed in 39]. In tumors that profit from high OXPHOS and high de novo fatty acid synthesis, PDK4 can have a protective effect. This seems to be the case for PC, which shows a high TCA cycle and OXPHOS activity [40, 41]. The prostate is an organ that is particularly dependent on high levels of citrate, which is the main component of the secreted prostatic fluid [40, 42]. In normal prostate, high levels of zinc prevent the TCA enzyme m-aconitase, thus preventing the conversion of citrate to isocitrate, truncating the TCA cycle and enabling the secretion of large amounts of citrate. In contrast, in PC cells the accumulation of zinc and secretion of citrate is decreased, and the TCA cycle and OXPHOS are increased in activity [40, 41]. These events are promoted by, for example, increased expression of aconitase in PC [39, 43, 44] and, based on our data, decreased expression of PDK4. Indeed, with siRNA experiments, we were able to show that PDK4 downregulation renders PC cells more metabolically active, while induced expression of PDK4 decreases both mitochondrial respiration and glycolysis rates in human PC cells.

Interestingly, during the course of this study, Oberhuber et al. [45] reported that gene expression of PDK4 is a promising independent prognostic marker in primary PC. They compared low STAT3 to high STAT3 primary PC at the transcriptomic and proteomic levels and found that gene expression of *PDK4* was significantly downregulated in low *STAT3* patients [45]. They analyzed the association of *PDK4* expression with biochemical recurrence and showed that low *PDK4* expression is significantly associated with a higher risk of biochemical recurrence and that *PDK4* predicts disease recurrence independent of ISUP grading in low‐/intermediate‐risk primary tumors. In addition, *PDK4* is an independent predictor of biochemical recurrence compared to ISUP grading and clinical staging, as well as pathological staging and pre‐surgical PSA levels in primary and metastatic tumors. We also noted that in primary PC, PDK4 expression is lower in higher Gleason grade tumors and that low PDK4 expression is associated with poorer recurrence-free survival of primary PC. Furthermore, we showed that levels of PDK4 are decreased in advanced PC using two data sets, our own for CRPC [31] and that of Taylor et al. [46] for both non-castrate and castration-resistant metastases. Collectively, these data show that PDK4 is a promising prognostic marker in PC, and a clinically relevant target of miR-32.

## Materials and methods

### Transgenic mice

All animal experimentation and care procedures were carried out in accordance with guidelines and regulations of the National Animal Experiment Board of Finland and were approved by the board of laboratory animal work of the State Provincial Offices of South Finland (license numbers ESAVI/6271/04.10.03/2011 and ESAVI/5147/04.10.07/2015). Generation of transgenic mir-32 mice in FVB/N background has been previously described in Latonen et al. [29]. Mice transgenic for oncogenic Myc (hiMyc mice) in FVB/N background have been previously described by Ellwood-Yen et al. [30]. DNA for genotyping was extracted from ear samples by overnight incubation at 55 °C in tissue lysis buffer (100 mM Tris pH 8, 300 mM NaCl, 10 mM EDTA) supplemented with 1% SDS and 200 ng/ml proteinase K, followed by standard EtOH precipitation. Genotyping was performed by qPCR using Maxima SYBRgreen (ThermoFischer Scientific Inc., Waltham, MA, USA) according to the manufacturer’s instructions. The primers used for genotyping have been previously described [29, 30].

### Mouse tissue samples and histology

Tissues were fixed either in formalin or in PAXgene™ (PreAnalytiX GmbH, Hombrechtikon, Switzerland) according to manufacturer’s recommendations, and embedded in paraffin. The prostate blocks were sectioned through as 5 µm-thick sections. The histology throughout the prostate was analyzed on hematoxylin and eosin (HE)-stained sections every 50 µm apart. Sections were whole slide imaged with Zeiss Axioskop40 microscope (Carl Zeiss MicroImaging, NY, USA) using a ×20 objective, a CCD color camera (QICAM Fast; QImaging, Canada), and a motorized specimen stage (Märzhäuser Wetzlar GmbH, Germany). The automated image acquisition was controlled by the Surveyor imaging system (Objective Imaging, UK). Uncompressed bitmap outputs were converted by JVSdicom Compressor application to JPEG2000 WSI format, and snapshot images were obtained through JVSView program and ImageJ software (version 1.52p) [47, 48]. Quantitation of tumor burden was performed in Cytomine (Version 1.3.5; Cytomine Corporation SA, Belgium) by annotating tumor areas with freehand selection tool on whole slide images of tissue sections taken every 50 µm apart throughout the organ and calculating sums of tumor areas of all sections for each prostate.

### Immunohistochemistry

Sections were deparaffinized and antigen retrieval was performed at +98 °C for 15 min in Tris-EDTA -buffer (pH 9), supplemented with 0.05% Tween-20. The staining was performed by Lab Vision Autostainer (ThermoFischer Scientific Inc.), using antibodies against cleaved Caspase-3 (Asp175, clone D3E9; Cell Signaling Technology, Danvers, MA, USA), Ki-67 (Sp6; ThermoFischer Scientific Inc.), and phosphorylated Histone H3 (Ser10; Cell Signaling Technology), followed by a secondary antibody (N-Histofine® Simple Stain MAX PO; Nichirei, Tokyo, Japan) and ImmPACT DAB (Vector Laboratories, Burlingame, CA, USA) as the chromogen. The sections were counterstained with hematoxylin and mounted with DPX mounting medium (Sigma-Aldrich), and digitized as described above. Assessment of cells positive for cleaved Caspase-3, Ki-67, and pH3 stainings was carried out with ImageJ cell counter. Between 500 and 3000 nuclei were counted per sample, and the number of antibody-stained positive nuclei relative to counterstained nuclei was calculated.

### RNA extraction

Tissues for RNA extraction were collected and stored in RNAlater® (ThermoFischer Scientific Inc.). RNA was extracted with manual homogenization by pressing a sample repeatedly through 20 G–22 G needles and using TriReagent® (Sigma-Aldrich) according to the manufacturer’s instructions. RNA from the tumorous prostate was extracted from tissues of 6-month-old mice. Prostates were fixed in PAXgene molecular fixative (PreAnalytiX GmbH) and embedded in paraffin. The prostate blocks were sectioned, and 10 × 5 µm-thick sections were used for RNA extraction with PAXgene Tissue RNA Kit (PreAnalytiX GmbH). Adjacent, HE-stained sections were used to confirm that the sections in RNA extractions contained a significant proportion of tumorous material. RNA extraction of clinical tumor samples used for RT-qPCR has been previously described [31].

### Microarray analysis

Global mRNA expression data were obtained using Agilent Mouse Gene Expression Array 44 K (Agilent Technologies, Santa Clara, CA, USA) according to the manufacturer’s protocols. In brief, the RNA from the clinical samples was labeled with Cy3 or Cy5 fluorochromes and subsequently co-hybridized for 21 h on Agilent 4X44K mouse gene expression array slides. Samples of 6-month-old hiMyc (*n* = 4) and miR-32xhiMyc (*n* = 4) mouse prostates were pooled for analysis. The slides were subsequently scanned on an Agilent C scanner and the raw scan data were extracted using the Agilent Feature Extraction software ver. 11.0.1.1 and normalization were performed to sample-wise means. Genes detected in both samples, and with an expression fold change over the threshold of 2 were considered significantly altered. The original data are submitted to Gene Expression Omnibus (GSE169323).

### RT-qPCR analysis

Quantitative RT-PCR for miRNAs was performed using TaqMan microRNA Assay (Applied Biosystems, Foster City, CA, USA) and the CFX96 q-RT-PCR detection system (Bio-Rad Laboratories Inc.) according to the manufacturers’ recommendations. miR-32 expression was normalized to RNU6B expression. Quantitative RT-PCR for assessing mRNA levels was performed by SYBRgreen method, using either *B-actin* or *TBP* as a reference gene. cDNA was made using Maxima RT reverse transcriptase (Thermo Fischer Scientific, Inc.). RT-qPCR reactions were performed on the CFX96 q-RT-PCR detection system (Bio-Rad Laboratories Inc., Hercules, CA, USA) or the LightCycler® 480 II system (Roche, Basel, Switzerland) using Maxima SYBR Green (Fermentas Inc., Burlington, ON, Canada) or Maxima SYBR Green/ROX qPCR Master Mix (Thermo Fischer Scientific). The primer sequences that were used are listed in Supplementary Table 6.

### Cell culture and transfections

22Rv1, PC-3, LNCaP, and HeLa cells (ATCC, Manassas, VA, USA) were maintained in recommended culture conditions. For esiRNA and pre-miRNA transfections, cells were reverse transfected with 20 nM of the targeting molecules using INTERFERin® transfection reagent (Polyplus Transfection SA, Illkirch, France) and following the manufacturer´s recommended transfection conditions and incubated for the indicated times. esiRNA targeting *PDK4* (heterogeneous pool of siRNA of natural RNA; Mission® esiRNA) was obtained from Merck KGaA (Darmstadt, Germany; EHU007651), and Mission® esiRNA targeting *GFP* (EHUEGFP) and siRNA targeting Firefly Luciferase (AM4629, Ambion, Thermo Fisher Scientific) were used as controls. miRNAs targeting miR-32-3p and -5p (Pre-miR miRNA Precursor, AM17100) were obtained from ThermoFischer Scientific (pre-miR-32-3p ID: PM12716; pre-miR-32-5p ID: PM12584), and Pre-miR^TM^ Negative Control (AM17110) was used as control. Plasmid transfections were performed using FuGENE® HD Transfection Reagent (Promega, Madison, WI, USA) with 100 ng of plasmid with 3:1 ratio of transfection reagent to DNA using the manufacturer’s recommended transfection conditions. Co-transfections were performed using Dharmacon^TM^ DharmaFECT^TM^ Duo Transfection reagent (Perkin Elmer, Inc., Waltham, MA, USA) with 20 nM miRNA and 100 ng plasmid using the manufacturer’s recommended transfection conditions.

### Luciferase assay

The luciferase assay was performed using Switch Gear Genomics LightSwitch^TM^ GoClone^®^ 3′-UTR reporter constructs (Active Motif, Carlsbad, CA, USA) and Pre-miR™ miRNA Precursor (Thermo Fisher Scientific, Inc.), according to the manufacturer´s recommendations. HeLa cells were seeded at >80% confluency in a white 96-well plate. The following day, the cells were co-transfected in triplicates with 100 ng of either GoClone empty reported vector, random-3′-UTR vector, or PDK4-3′-UTR vector and with either 20 nM pre-miR-32-3p, pre-miR-32-5p, or pre-miR control using DharmaFECT Duo transfection reagent (Perkin Elmer, Inc. Waltham, MA, USA). The following day, 100 µL of LightSwitch^TM^ assay buffer supplemented with assay substrate was added to each well and the luciferase signal was measured after 30 min of incubation at RT using a Luminoskan™ Ascent Microplate Luminometer (ThermoFischer Scientific, Inc.).

### Metabolic assays

Cell metabolic analysis was performed using Alamar Blue reagent (Thermo Fischer Scientific) according to the manufacturer’s instructions. 22Rv1 cells were transfected with esiRNA using INTERFERin® as above and metabolic activity was analyzed after 5 days.

Mitochondrial respiration and glycolysis rate were assessed using the Seahorse XFe96 analyzer (Agilent Technologies). PC-3 cells were seeded in complete growth medium on Agilent XF96 cell culture microplate at a density of 2 × 10^4^ cells per well and transfected the following day with 100 ng of either control plasmid (pcDNA3.1+, Thermo Fischer Scientific, Inc.) or PDK4 expression plasmid (Origene, Rockville, MD, USA. SC118542) using FuGENE® HD Transfection Reagent (Promega Corporation, Madison, WI, USA) with a 3:1 ratio of DNA:FuGENE. After 5 days, the medium was changed to Seahorse XF Dulbecco’s Modified Eagle Medium (DMEM) medium, pH 7.4, supplemented with 1 mM pyruvate, 2 mM glutamine, and 10 mM glucose and equilibrated for 1 h at 37 °C in a non-CO_2_ incubator before the experiment. Analysis was performed utilizing Mitochondrial Stress Test Kit (Agilent Technologies) and Glycolysis Rate Kit (Agilent Technologies). For the mitochondrial function assay, 1 µM oligomycin, 0.5 µM FCCP, and 0.5 µM rotenone/antimycin A were used. For the glycolysis measurement, 0.5 µM rotenone/antimycin A and 50 mM 2-deoxyglucose were used. The results of oxygen consumption rate and extracellular acidification rate were normalized based on the total protein level in each well of the microplate.

### Growth assay

PC-3 were plated at 40% confluency in 96-well plate and transfected with nine replicates the following day with FuGENE HD® Transfection reagent as above. Transfected cells were monitored by phase-contrast imaging every 8 h for the indicated times using IncuCyte® S3 Live-Cell Analysis system (Sartorius AG, Göttingen, Germany). Analysis of cell confluence was performed with automated analysis in IncuCyte 2021B (Sartorius AG).

### Immunocytochemistry

PC-3 cells were seeded on coverslips and transfected with FuGENE HD® as above. After incubation for 3 days, cells were fixed in 4% paraformaldehyde for 30 min at RT and permeabilized with 0.5% NP40 in phosphate-buffered saline (PBS). Anti-PDK4 primary antibody (1:100, PA5-13776, Thermo Fischer Scientific) and Alexa Fluor^TM^ 568 goat-anti-rabbit (Thermo Fischer Scientific) secondary antibody were used. Nuclei were stained using DAPI. Cells were imaged using Zeiss LSM800 confocal microscope (Zeiss, Jena, Germany).

### Western blotting

Cells were lysed in Triton-X lysis buffer containing 50 mM Tris-HCl, pH 7.5, 150 mM NaCl, 0.5% Triton-X-100, 1 mM phenylmethylsulfonyl fluoride, 1 mM DTT, and 1 × Complete protease inhibitor cocktail (Roche), after which cellular debris was removed by centrifugation. Samples were resuspended in 4× Laemmli sample buffer and denatured at 95 °C. Proteins were separated by 10% sodium dodecyl sulfate–polyacrylamide gel electrophoresis (SDS-PAGE) gel and immobilized onto Nitrocellulose-membranes (Thermo Fisher Scientific). Primary antibodies against PDK4 (PA5-13776, Thermo Fischer Scientific), and actin (ACTN05 C4, Abcam, Cambridge, UK), were used together with anti-mouse or anti-rabbit HRP-conjugated antibodies produced in goats (Invitrogen, Thermo Fisher Scientific). Chemiluminescence reactions were generated using Clarity Western ECL Substrate reagent (Bio-Rad Laboratories) and measured using ChemiDoc MP Imaging system (Bio-Rad Laboratories). Quantitation of signals was performed using Image Lab software (Version 6.0.0 build 25, Bio-Rad Laboratories).

### In silico data analysis

Functional classification of the altered genes was performed with The PANTHER (Protein ANalysis THrough Evolutionary Relationships) Classification System (http://pantherdb.org/; version 11.1) [49]. For the target predictions for mm-miR-32, Targetscan (Targetscan.org) [50] platform was utilized. Gene set enrichment and pathway analyses were performed with online tools of DAVID (https://david.ncifcrf.gov) [51], KEGG (https://www.kegg.jp) [52], and Enrichr (https://maayanlab.cloud/Enrichr) [53]. RNA expression data previously generated by us (Tampere cohort) [31] and others (GSE21032 [46]; GSE25183 [54]), were queried for PDK4 expression in human tissue samples and cell lines.

### Statistical analyses

Statistical analyses were performed with GraphPad Prism statistics software (version 5.03; GraphPad Software Inc., La Jolla, CA, USA. Differences in prostate size, tumor nuclear density, parameters retrieved from IHC labeling assays, gene expression in cellular assays, metabolic assays, and luciferase assays were assessed by two-tailed *t* test. The significance of differences in mouse tumor burden and gene expression analyses were evaluated by Mann–Whitney *U* test. Gene expression differences in clinical sample data sets were determined by Kruskal–Wallis or Mann–Whitney *U* test, and Spearman correlation analysis was applied to gene expression correlation assessment. The significance of the difference in survival was assessed by Mantel–Cox log-rank test and in cell growth curves with two-way analysis of variance.

## Supplementary information

Supplementary legends

Supplementary figures

Supplementary tables
