# Supplementary material for: miR-32 promotes MYC-driven prostate cancer
Source: Oncogenesis. 2022 Mar 1;11(1):11. doi: 10.1038/s41389-022-00385-8 (PMC8885642; doi:10.1038/s41389-022-00385-8)
Supplement: Supplementary file 1 — Supplementary legends [file 41389_2022_385_MOESM1_ESM.docx]

**Supplementary figure legends**

**Supplementary Figure 1. Verification of expression of transgenes.** RT-qPCR analysis of prostate tissue. A. Expression of miR-32 in ventral, lateral and dorsal prostates of mice at 3 months. B. Expression levels of miR-32 in wt, miR-32 transgenic, hiMYC, and miR-32xhiMYC mice at 1 month. C. Expression of MYC in ventral, lateral and dorsal prostates of mice at 3 months. D. Expression levels of MYC in wt, miR-32 transgenic, hiMYC, and miR-32xhiMYC mice at 1 month.

**Supplementary Figure 2. Histopathological analysis of effects of transgenic miR-32 expression in hiMYC-induced prostate cancer in mouse.** A. HE-stained histological sections in miR-32xhiMYC mice at 3 months of age showing signs of local invasion (arrows) perivascularly (left panel) and outside the basal smooth muscle layer surrounding prostate glands (right panel). A vein (black asterisk) and basal smooth muscle layer of a prostatic gland (white asterisk) are marked. C. Percentage of prostate epithelial cells at 1 months (left panel) and prostate tumor cells at 6 months (right panel) of age positive for cleaved caspase-3 immunostaining in hiMYC and miR-32xhiMYC mice.

**Supplementary Figure 3. Pathway analysis of genes found differentially expressed in miR-32xhiMYC mouse prostate.** Differential expression of genes between miR-32xhiMYC and hiMYC prostates with tumors in mice at 6 months of age in microarray analysis. A. Enriched KEGG pathways based on mouse genes. B. Enriched WikiPathway pathways based on mouse genes. C. Enriched KEGG pathways based on human homologs. D. Enriched WikiPathway pathways based on human homologs.

**Supplementary Figure 4. Expression analysis of PDK4 in mouse prostate and prostate-derived cell lines.** A. RT-qPCR validation of microarray analysis results in miR-32xhiMYC and hiMYC prostates with tumors in mice at 6 months of age showing that Pdk4 expression is downregulated by transgenic miR-32 expression. B. Expression of miR-32 and PDK4 mRNA in the dataset of Taylor *et al.* [46] show statistically significant inverse correlation. C. Expression analysis of PDK4 in human prostate-derived normal and cancer cell lines in the dataset of Presner *et al.* [49]. D. siRNA downregulation of PDK4 mRNA levels in 22Rv1 PC cells. E. Downregulation of PDK4 protein levels in 22Rv1 cells upon siRNA targeting. Asterisks mark non-specific bands in the western blot. Quantitation of PDK4 signals are shown. Actin shown as loading control. Error bars, standard deviation. ***p-value <0.001.

**Supplementary Tables S1-S6.**
