# Supplementary figures and images for: miR-32 promotes MYC-driven prostate cancer
Source: Oncogenesis. 2022 Mar 1;11(1):11. doi: 10.1038/s41389-022-00385-8 (PMC8885642; doi:10.1038/s41389-022-00385-8)

## Supplementary Figure 1

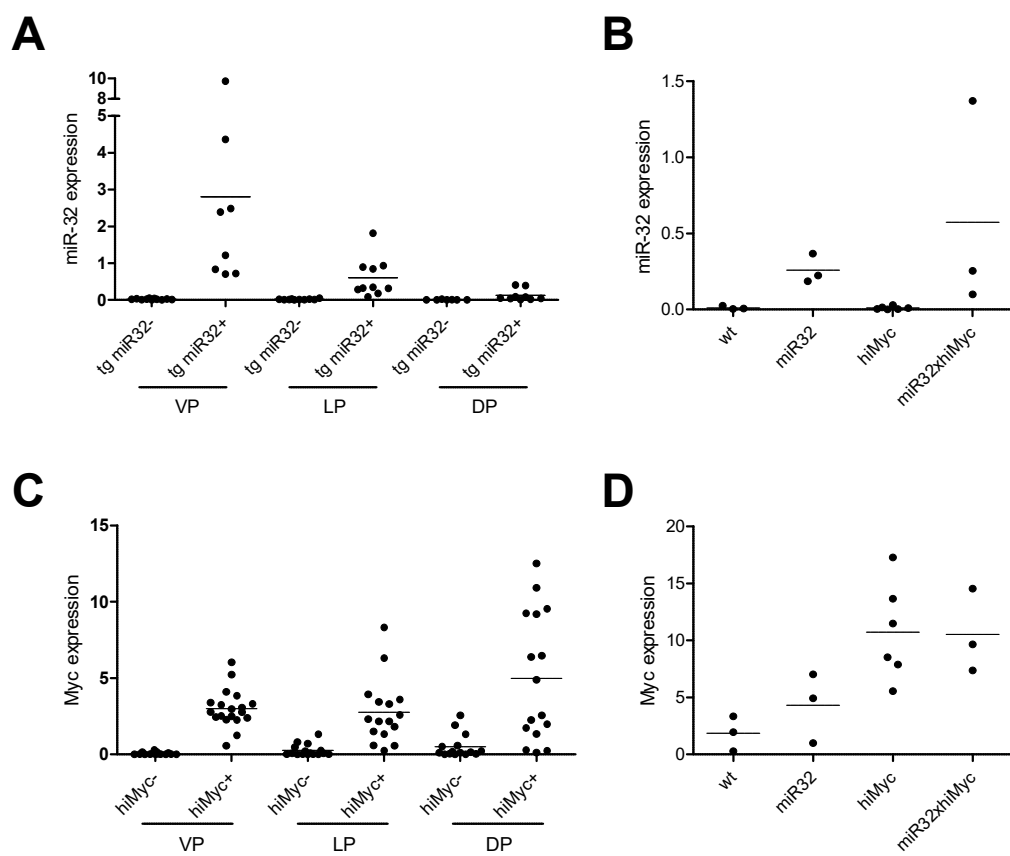

## Supplementary Figure 2

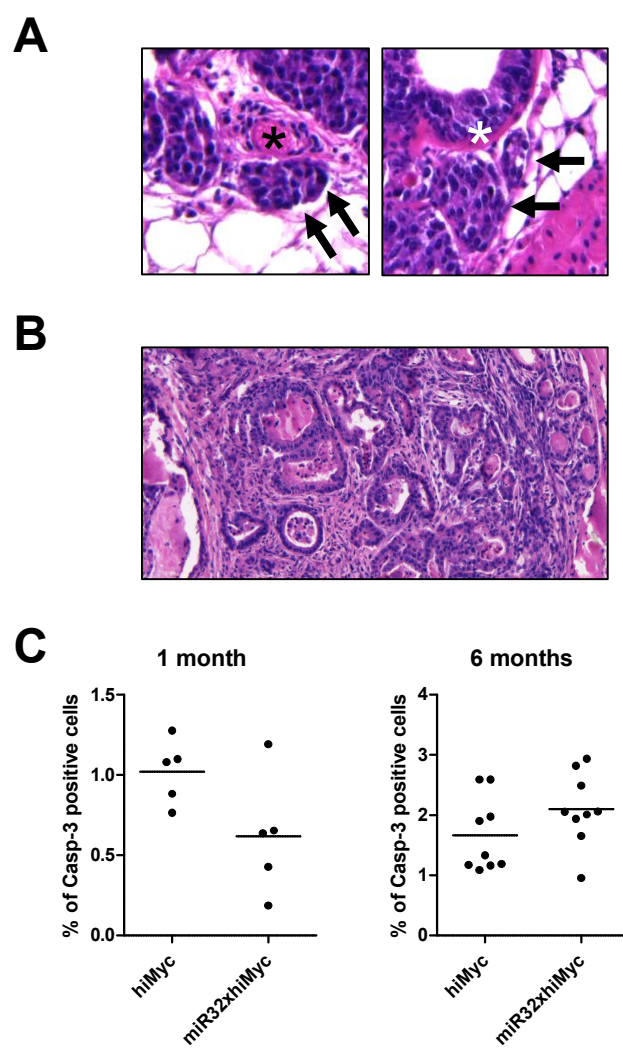

# Supplementary Figure 3

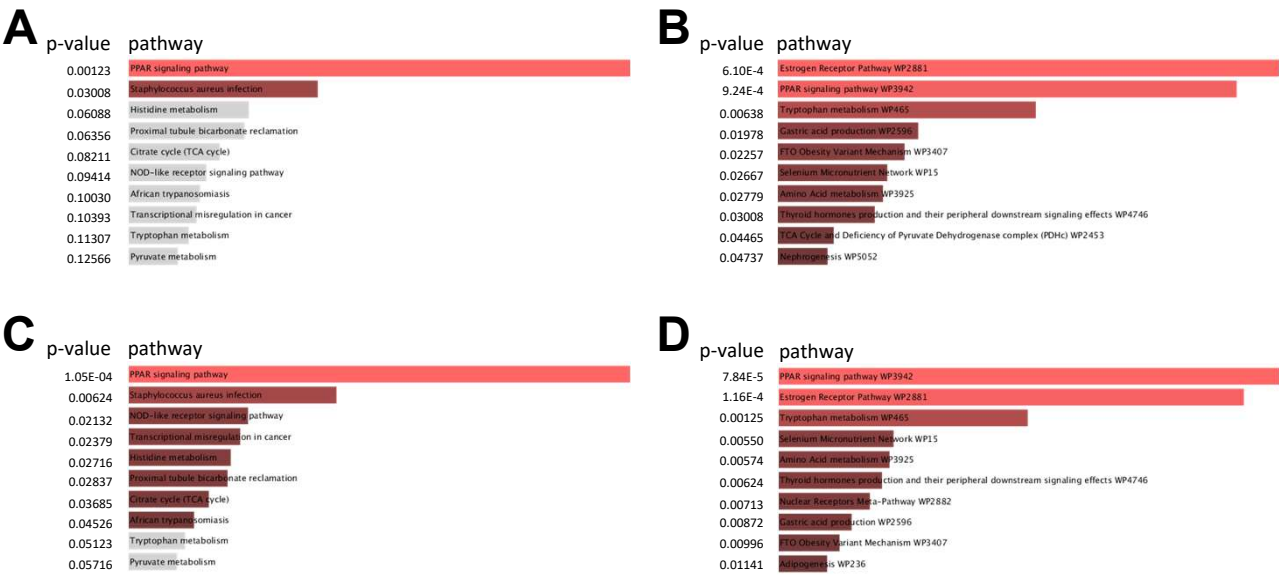

# Supplementary Figure 4

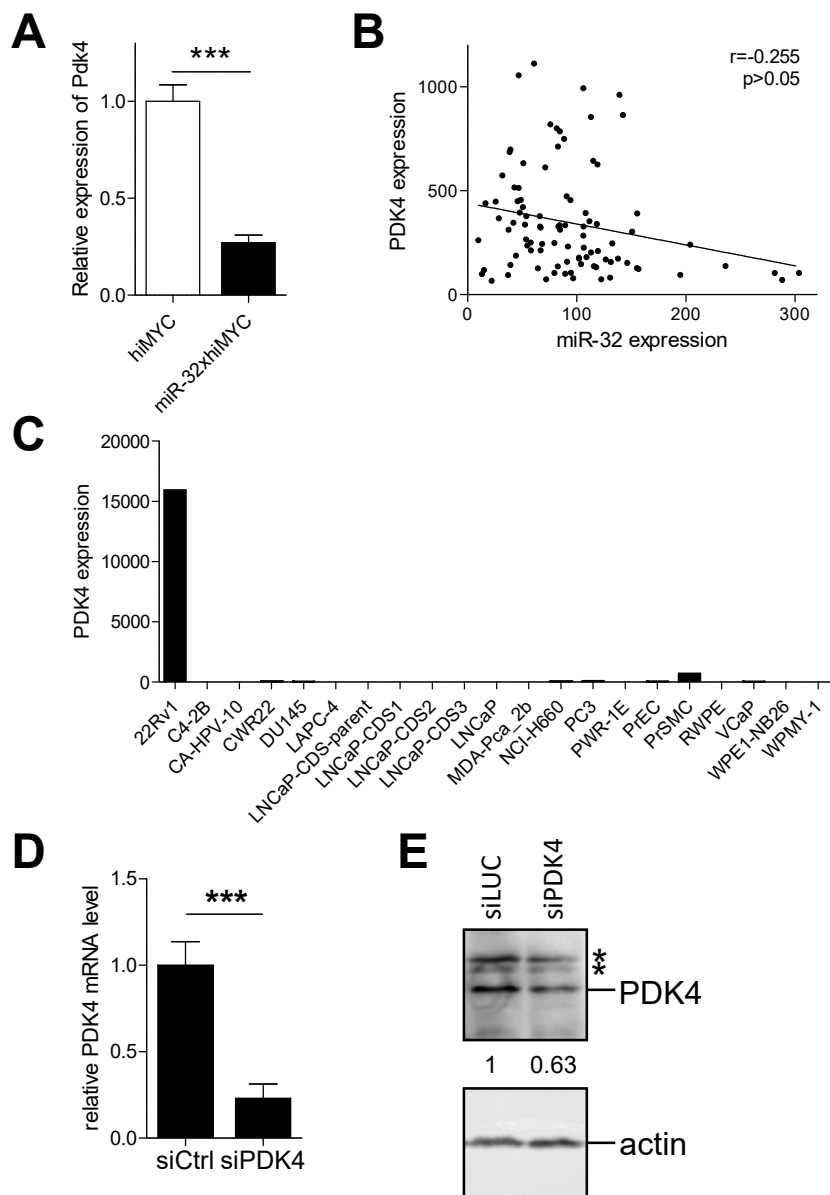

Supplement: Supplementary file 2 — Supplementary figures [file 41389_2022_385_MOESM2_ESM.pdf]
