# Supplementary material for: miR-32 promotes MYC-driven prostate cancer
Source: Oncogenesis. 2022 Mar 1;11(1):11. doi: 10.1038/s41389-022-00385-8 (PMC8885642; doi:10.1038/s41389-022-00385-8)
Supplement: Supplementary file 3 — Supplementary tables [file 41389_2022_385_MOESM3_ESM.pdf]

Supplementary Table 1. Differentially expressed genes in miR-32xhiMYC compared to hiMYC mouse prostates.

GeneSymbol	logFC	AveExpr	P.Value	adj.P.Val	ProbeID	prediced miR-32 target (5p/3p)	downregulated in miR-32 over-expressing prostates (Latonen et al. 2017)
Ucp1	-2,25	7,56342	5,9E-18	6,124E-14	A_51_P426353		
Cidea	-2,073	9,15231	3,2E-15	1,228E-12	A_51_P199168		
Pck1	-1,544	6,83912	1,1E-17	6,124E-14	A_66_P121110	3p	
Fabp4	-1,533	11,1287	7E-17	1,166E-13	A_51_P336833	3p	x
Akr1b7	-1,458	6,7299	1,8E-14	3,971E-12	A_51_P331288		
Car6	-1,445	8,244	1,6E-13	2,009E-11	A_55_P2004370		
Spink5	-1,29	11,0711	2,2E-17	6,124E-14	A_66_P126147		x
Smgc	-1,28	8,85876	8,7E-15	2,383E-12	A_55_P1958720		
Spink1	-1,146	14,0755	2,6E-17	6,124E-14	A_51_P365516		x
Mup19	-1,113	11,0319	4,1E-14	7,22E-12	A_55_P1974080		
Mup4	-1,1	12,1274	2,3E-15	1,038E-12	A_55_P2010093		
Pdk4	-1,068	7,26241	6E-11	1,469E-09	A_51_P350453	3p	
Msmb	-1,066	11,161	1E-13	1,471E-11	A_52_P125636		
Mup-ps16	-1,066	12,6684	5,7E-17	1,04E-13	A_55_P1981195		
Serinc4	-1,06	8,32246	0,02932	0,0378961	A_55_P2050997		
Mup20	-1,054	10,9512	1,1E-16	1,296E-13	A_55_P2063654		
Mup6	-1,046	8,07104	7,9E-13	5,958E-11	A_55_P2061104		
Mup1	-1,031	9,21374	3,5E-14	6,478E-12	A_55_P1979904		
Xlr3b	-1,007	7,96109	5,3E-13	4,47E-11	A_55_P1959683		
Cfap53	1,01	7,25549	7,4E-13	5,733E-11	A_55_P2063715		
Dio3	1,014	6,82664	1,7E-10	3,239E-09	A_55_P2091468		
Gzmd	1,028	6,11665	2,3E-14	4,548E-12	A_52_P554650		
3930401B19Rik	1,051	13,3211	1,2E-15	7,293E-13	A_55_P1957213		
Defa-ps1	1,061	7,03372	8,5E-14	1,289E-11	A_55_P2054373		
Gm7714	1,076	9,76678	1,1E-15	7,293E-13	A_55_P2062078		
Sema3a	1,085	6,48689	1,7E-13	2,057E-11	A_55_P2054013	5p/3p	
Defa-rs12	1,096	7,48532	2,8E-14	5,394E-12	A_55_P2045213		
Ceacam10	1,11	6,76861	4,4E-11	1,174E-09	A_52_P134195		x
Saa2	1,143	8,24441	1,2E-15	7,293E-13	A_55_P1994807		
Srgn	1,15	9,45918	5,2E-16	5,05E-13	A_55_P2094925	3p	x
Ang3	1,167	7,4931	2,3E-09	2,336E-08	A_51_P179504		
Scgb2b20	1,19	10,3842	1,2E-15	7,293E-13	A_55_P2057004		
Slc38a5	1,192	8,54768	3,2E-17	6,461E-14	A_55_P2010641		
Svs1	1,26	6,37915	2,3E-14	4,548E-12	A_55_P2079231		
Defa-rs2	1,29	7,76676	3,5E-14	6,478E-12	A_55_P2018869		
Defa-rs4	1,312	7,43579	1,2E-15	7,293E-13	A_55_P2018865		
Saa3	1,361	7,19231	4,3E-15	1,475E-12	A_55_P1953169		x
Cpxm1	1,365	10,0665	2,8E-15	1,145E-12	A_51_P139108		
Dmkn	1,379	7,73831	9,9E-16	7,293E-13	A_55_P2133405		
Aldh1a2	1,424	9,68642	1,4E-13	1,828E-11	A_52_P58145		
Scgb2b27	1,452	8,66627	1,8E-17	6,124E-14	A_55_P2094373		
Gzmc	1,594	6,82393	9,8E-16	7,293E-13	A_55_P2135526		
Ctla2b	1,602	12,9436	1,9E-17	6,124E-14	A_51_P489522		
Aoc1	1,636	7,11017	9,4E-13	6,745E-11	A_51_P212068		
Slpi	1,641	9,4779	2,3E-14	4,577E-12	A_52_P472324		x
Defa4	1,691	8,5586	1,1E-14	2,842E-12	A_51_P497255		
Gm15292	1,75	9,33962	2,1E-14	4,459E-12	A_55_P2036898		
Defa1	1,779	9,97495	1,5E-13	1,872E-11	A_55_P1999110		
Defa36	1,779	9,25917	2,7E-15	1,145E-12	A_55_P1995018		
Scgb2b2	1,783	11,5864	2,6E-16	2,75E-13	A_55_P2061432		
Ctla2a	1,787	14,2635	2,3E-14	4,548E-12	A_55_P1955656		
Kng1	1,883	7,78146	5E-15	1,564E-12	A_55_P2003410		
Svs6	2,033	7,1215	7,9E-16	6,576E-13	A_51_P385030		
Gzmg	2,807	7,2924	2,4E-13	2,517E-11	A_55_P2060376		
Svs5	3,147	9,22819	2,7E-17	6,124E-14	A_51_P353162		
Grp	3,175	9,77423	1,4E-17	6,124E-14	A_51_P356055	3p	
Gzme	3,37	7,81136	1,9E-16	2,188E-13	A_55_P1971010		

Supplementary Table 2. Pathway analysis of gene expression in miR32 transgene-expressing compared to control prostates with hiMyc-induced tumors.
Enricher analysis of KEGG pathways based on significantly regulated mouse genes (55 genes).

Term	Overlap	P-value	Adjusted P-value	Genes
PPAR signaling pathway	3/74	0,001233	0,044374	FABP4;UCP1;PCK1
Staphylococcus aureus infection	2/95	0,030088	0,417251	DEFA4;DEFA1
Histidine metabolism	1/22	0,06089	0,417251	AOC1
Proximal tubule bicarbonate reclamation	1/23	0,063569	0,417251	PCK1
Citrate cycle (TCA cycle)	1/30	0,082116	0,417251	PCK1
NOD-like receptor signaling pathway	2/181	0,094148	0,417251	DEFA4;DEFA1
African trypanosomiasis	1/37	0,100301	0,417251	KNG1
Transcriptional misregulation in cancer	2/192	0,103933	0,417251	DEFA4;DEFA1
Tryptophan metabolism	1/42	0,113074	0,417251	AOC1
Pyruvate metabolism	1/47	0,125668	0,417251	PCK1
Arginine and proline metabolism	1/50	0,133141	0,417251	AOC1
Regulation of lipolysis in adipocytes	1/55	0,145455	0,417251	FABP4
Glycolysis / Gluconeogenesis	1/67	0,174312	0,417251	PCK1
Retinol metabolism	1/68	0,176674	0,417251	ALDH1A2
Adipocytokine signaling pathway	1/69	0,179028	0,417251	PCK1
Complement and coagulation cascades	1/85	0,215812	0,417251	KNG1
GABAergic synapse	1/89	0,224752	0,417251	SLC38A5
Inflammatory mediator regulation of TRP channels	1/98	0,244503	0,417251	KNG1
Chagas disease	1/102	0,253122	0,417251	KNG1
Neuroactive ligand-receptor interaction	2/341	0,253763	0,417251	GRP;KNG1
Glucagon signaling pathway	1/107	0,263759	0,417251	PCK1
Insulin resistance	1/108	0,265869	0,417251	PCK1
Sphingolipid signaling pathway	1/119	0,288686	0,417251	KNG1
AMPK signaling pathway	1/120	0,290725	0,417251	PCK1
Thyroid hormone signaling pathway	1/121	0,292759	0,417251	DIO3
FoxO signaling pathway	1/131	0,312783	0,417251	PCK1
Insulin signaling pathway	1/137	0,324528	0,417251	PCK1
Apelin signaling pathway	1/137	0,324528	0,417251	UCP1
cGMP-PKG signaling pathway	1/167	0,380362	0,472174	KNG1
Axon guidance	1/182	0,406553	0,487864	SEMA3A
Diabetic cardiomyopathy	1/203	0,441402	0,512596	PDK4
Regulation of actin cytoskeleton	1/218	0,465055	0,523187	KNG1
Thermogenesis	1/232	0,486241	0,530445	UCP1
Huntington disease	1/306	0,585251	0,619677	UCP1
PI3K-Akt signaling pathway	1/354	0,639179	0,657441	PCK1
Pathways in cancer	1/531	0,784755	0,784755	KNG1

Supplementary Table 3. Pathway analysis of gene expression in miR32 transgene-expressing compared to control prostates with hiMyc-induced tumors.
Enricher analysis of KEGG pathways based on human orthologs of significantly regulated mouse genes (25 genes)

**Term**	**Overlap**	**P-value**	**Adjusted P-value**	**Genes**
PPAR signaling pathway	3/74	1,05E-04	0,003797	FABP4;UCP1;PCK1
Staphylococcus aureus infection	2/95	0,006238	0,112281	DEFA4;DEFA1
NOD-like receptor signaling pathway	2/181	0,021317	0,17024	DEFA4;DEFA1
Transcriptional misregulation in cancer	2/192	0,023795	0,17024	DEFA4;DEFA1
Histidine metabolism	1/22	0,027156	0,17024	AOC1
Proximal tubule bicarbonate reclamation	1/23	0,028373	0,17024	PCK1
Citrate cycle (TCA cycle)	1/30	0,036854	0,186176	PCK1
African trypanosomiasis	1/37	0,045264	0,186176	KNG1
Tryptophan metabolism	1/42	0,051228	0,186176	AOC1
Pyruvate metabolism	1/47	0,057156	0,186176	PCK1
Arginine and proline metabolism	1/50	0,060696	0,186176	AOC1
Regulation of lipolysis in adipocytes	1/55	0,066567	0,186176	FABP4
Neuroactive ligand-receptor interaction	2/341	0,06723	0,186176	GRP;KNG1
Glycolysis / Gluconeogenesis	1/67	0,080514	0,186343	PCK1
Retinol metabolism	1/68	0,081667	0,186343	ALDH1A2
Adipocytokine signaling pathway	1/69	0,082819	0,186343	PCK1
Complement and coagulation cascades	1/85	0,101061	0,20281	KNG1
GABAergic synapse	1/89	0,105567	0,20281	SLC38A5
Inflammatory mediator regulation of TRP channels	1/98	0,115625	0,20281	KNG1
Chagas disease	1/102	0,120061	0,20281	KNG1
Glucagon signaling pathway	1/107	0,125576	0,20281	PCK1
Insulin resistance	1/108	0,126674	0,20281	PCK1
Sphingolipid signaling pathway	1/119	0,138675	0,20281	KNG1
AMPK signaling pathway	1/120	0,139758	0,20281	PCK1
Thyroid hormone signaling pathway	1/121	0,14084	0,20281	DIO3
FoxO signaling pathway	1/131	0,151587	0,203107	PCK1
Insulin signaling pathway	1/137	0,157972	0,203107	PCK1
Apelin signaling pathway	1/137	0,157972	0,203107	UCP1
cGMP-PKG signaling pathway	1/167	0,189215	0,234888	KNG1
Axon guidance	1/182	0,204416	0,245299	SEMA3A
Diabetic cardiomyopathy	1/203	0,225238	0,261567	PDK4
Regulation of actin cytoskeleton	1/218	0,23979	0,269764	KNG1
Thermogenesis	1/232	0,253135	0,276147	UCP1
Huntington disease	1/306	0,320018	0,338842	UCP1
PI3K-Akt signaling pathway	1/354	0,360285	0,370579	PCK1
Pathways in cancer	1/531	0,48989	0,48989	KNG1

Supplementary Table 4. Pathway analysis of gene expression in miR32 transgene-expressing compared to control prostates with hiMyc-induced tumors.
Enricher analysis of WikiPathway pathways based on significantly regulated mouse genes (55 genes).

Term	Overlap	P-value	Adjusted P-value	Genes
Estrogen Receptor Pathway WP2881	2/13	6,10E-04	0,015243	PDK4;PCK1
PPAR signaling pathway WP3942	3/67	9,24E-04	0,015243	FABP4;UCP1;PCK1
Tryptophan metabolism WP465	2/42	0,006386	0,070251	AOC1;ALDH1A2
Gastric acid production WP2596	1/7	0,019783	0,124112	GRP
FTO Obesity Variant Mechanism WP3407	1/8	0,022578	0,124112	UCP1
Selenium Micronutrient Network WP15	2/89	0,026675	0,124112	DIO3;SAA2
Amino Acid metabolism WP3925	2/91	0,027794	0,124112	PDK4;PCK1
Thyroid hormones production and their peripheral downstre	2/95	0,030088	0,124112	UCP1;DIO3
TCA Cycle and Deficiency of Pyruvate Dehydrogenase comple	1/16	0,044654	0,125075	PCK1
Nephrogenesis WP5052	1/17	0,047379	0,125075	ALDH1A2
ACE Inhibitor Pathway WP554	1/17	0,047379	0,125075	KNG1
Cells and molecules involved in local acute inflammatory resp	1/17	0,047379	0,125075	KNG1
Fatty acid transporters WP5061	1/18	0,050096	0,125075	FABP4
Adipogenesis WP236	2/130	0,053062	0,125075	UCP1;PCK1
Galanin receptor pathway WP4970	1/21	0,058203	0,128046	UCP1
Nuclear Receptors Meta-Pathway WP2882	3/319	0,062643	0,129202	SRGN;PDK4;PCK1
Differentiation of white and brown adipocyte WP2895	1/25	0,068905	0,133757	CIDEA
RAS and bradykinin pathways in COVID-19 WP4969	1/29	0,079488	0,145729	KNG1
Ethanol effects on histone modifications WP3996	1/31	0,084736	0,147172	ALDH1A2
Vitamin A and carotenoid metabolism WP716	1/43	0,115607	0,189596	ALDH1A2
Glycolysis and Gluconeogenesis WP534	1/45	0,120652	0,189596	PCK1
Vitamin B12 metabolism WP1533	1/50	0,133141	0,199711	SAA2
Complement and Coagulation Cascades WP558	1/58	0,152761	0,219179	KNG1
Folate Metabolism WP176	1/69	0,179028	0,239416	SAA2
Glucocorticoid Receptor Pathway WP2880	1/70	0,181376	0,239416	SRGN
Electron Transport Chain (OXPHOS system in mitochondria) \	1/103	0,255261	0,323985	UCP1
Thermogenesis WP4321	1/108	0,265869	0,324951	UCP1
22q11.2 copy number variation syndrome WP4657	1/131	0,312783	0,358168	ALDH1A2
Angiopoietin Like Protein 8 Regulatory Pathway WP3915	1/132	0,314754	0,358168	PCK1
Male infertility WP4673	1/146	0,341778	0,375956	MSMB
Vitamin D Receptor Pathway WP2877	1/182	0,406553	0,421018	KNG1
Ciliopathies WP4803	1/183	0,40826	0,421018	CFAP53
PI3K-Akt signaling pathway WP4172	1/340	0,624205	0,624205	PCK1

Supplementary Table 5. Pathway analysis of gene expression in miR32 transgene-expressing compared to control prostates with hiMyc-induced tumors.
Enricher analysis of WikiPathway pathways based on human orthologs of significantly regulated mouse genes (25 genes)

Term	Overlap	P-value	Adjusted P-value	Genes
PPAR signaling pathway WP3942	3/67	7,84E-05	0,001914	FABP4;UCP1;PCK1
Estrogen Receptor Pathway WP2881	2/13	1,16E-04	0,001914	PDK4;PCK1
Tryptophan metabolism WP465	2/42	0,001253	0,013778	AOC1;ALDH1A2
Selenium Micronutrient Network WP15	2/89	0,005496	0,033605	DIO3;SAA2
Amino Acid metabolism WP3925	2/91	0,005738	0,033605	PDK4;PCK1
Thyroid hormones production and their peripheral downstre	2/95	0,006238	0,033605	UCP1;DIO3
Nuclear Receptors Meta-Pathway WP2882	3/319	0,007128	0,033605	SRGN;PDK4;PCK1
Gastric acid production WP2596	1/7	0,008718	0,035964	GRP
FTO Obesity Variant Mechanism WP3407	1/8	0,009958	0,036513	UCP1
Adipogenesis WP236	2/130	0,011406	0,03764	UCP1;PCK1
TCA Cycle and Deficiency of Pyruvate Dehydrogenase comple	1/16	0,019821	0,048998	PCK1
Nephrogenesis WP5052	1/17	0,021047	0,048998	ALDH1A2
ACE Inhibitor Pathway WP554	1/17	0,021047	0,048998	KNG1
Cells and molecules involved in local acute inflammatory resp	1/17	0,021047	0,048998	KNG1
Fatty acid transporters WP5061	1/18	0,022272	0,048998	FABP4
Galanin receptor pathway WP4970	1/21	0,025937	0,053495	UCP1
Differentiation of white and brown adipocyte WP2895	1/25	0,030804	0,059795	CIDEA
RAS and bradykinin pathways in COVID-19 WP4969	1/29	0,035647	0,065353	KNG1
Ethanol effects on histone modifications WP3996	1/31	0,03806	0,066104	ALDH1A2
Vitamin A and carotenoid metabolism WP716	1/43	0,052416	0,086097	ALDH1A2
Glycolysis and Gluconeogenesis WP534	1/45	0,054789	0,086097	PCK1
Vitamin B12 metabolism WP1533	1/50	0,060696	0,091043	SAA2
Complement and Coagulation Cascades WP558	1/58	0,070073	0,100539	KNG1
Folate Metabolism WP176	1/69	0,082819	0,11084	SAA2
Glucocorticoid Receptor Pathway WP2880	1/70	0,08397	0,11084	SRGN
Electron Transport Chain (OXPHOS system in mitochondria) V	1/103	0,121167	0,153788	UCP1
Thermogenesis WP4321	1/108	0,126674	0,154824	UCP1
22q11.2 copy number variation syndrome WP4657	1/131	0,151587	0,17371	ALDH1A2
Angiopoietin Like Protein 8 Regulatory Pathway WP3915	1/132	0,152654	0,17371	PCK1
Male infertility WP4673	1/146	0,167464	0,184211	MSMB
Vitamin D Receptor Pathway WP2877	1/182	0,204416	0,211839	KNG1
Ciliopathies WP4803	1/183	0,20542	0,211839	CFAP53
PI3K-Akt signaling pathway WP4172	1/340	0,348783	0,348783	PCK1

Supplementary table 6. Primers used in qRT-PCR assays.

Target gene	Forward primer	Reverse primer	Annealing T
Ang3	CGAAGCTAGACACATCCCCC	TCTGTAATCCCGGCCAGTTG	64
B-actin	CGAGCGGTTCCGATGCCCTG	ACGCAGCTCAGTAACAGTCCGC	65
Dio3	TCAGACGACAACCGTCTGTG	AAAATTGAGCACCAACGGGC	60
Fabp4	GATGAAATCACCGCAGACGAC	AAACTCTTGTGGAAGTCACGC	62
Msmb	GGAAGCTTGGCTGGGCAGTCT	GGAAGCTTGGCTGGGCAGTCT	65
MYC	ACCAGAGTTTCATCTGCGACC	GGGTCGATGCACTCTGAGG	62
Pdk4	TGAACACTCCTTCGGTGCAG	GCCTTGAGCCATTGTAGGGA	58
Spink1	TTCGACAATGAAGGTGGCTGT	CACTGCATCATGGCAACTAGC	60
Spink5	GAGTTCCAGTGGTGGGAACC	CCCGAGTGCAGAGGAGTTTC	65
Srgn	ATCGAGGAGAAGGGACCACA	CCCGAACCTGACCCATAGTC	62
